# Bis(2-methyl-1*H*-imidazol-3-ium) naphthalene-1,5-disulfonate dihydrate

**DOI:** 10.1107/S1600536812019149

**Published:** 2012-05-05

**Authors:** Yu-feng Wang

**Affiliations:** a Ordered Matter Science Research Center, Southeast University, Nanjing 211189, People’s Republic of China

## Abstract

The asymmetric unit of the title organic salt, 2C_4_H_7_N_2_^+^·C_10_H_6_O_6_S_2_^2−^·2H_2_O, consists of a 2-methyl­imidazolium cation, a half of a naphthalene-1,5-disulfonate anion, which lies about a center of symmetry, and a water mol­ecule. In the crystal, N—H⋯O and O—H⋯O hydrogen bonds link the cations, anions and water mol­ecules into the layers parallel to (111).

## Related literature

For general background to dielectric–ferroelectric phase transitions, see: Ye *et al.* (2009 ▶); Zhang *et al.* (2009 ▶). For the structures of naphthalene-1,5-disulfonate salts with *N*-heterocyclic cations, see: Janczak & Perpétuo (2008 ▶); Wang *et al.* (2008 ▶).
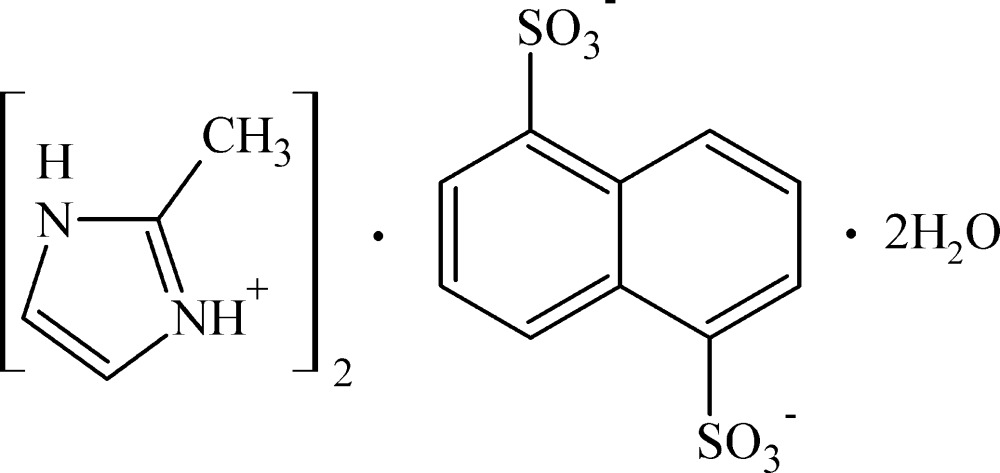


## Experimental

### 

#### Crystal data

2C_4_H_7_N_2_^+^·C_10_H_6_O_6_S_2_^2−^·2H_2_O
*M* *_r_* = 488.53
Triclinic, 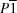

*a* = 7.1301 (14) Å
*b* = 8.1773 (16) Å
*c* = 9.970 (2) Å
α = 75.58 (3)°
β = 75.10 (3)°
γ = 80.34 (3)°
*V* = 540.7 (2) Å^3^
*Z* = 1
Mo *K*α radiation
μ = 0.30 mm^−1^
*T* = 293 K
0.23 × 0.22 × 0.18 mm

#### Data collection

Rigaku SCXmini diffractometer
Absorption correction: multi-scan (*CrystalClear*; Rigaku, 2005 ▶) *T*_min_ = 0.933, *T*_max_ = 0.947
5695 measured reflections
2475 independent reflections
1490 reflections with *I* > 2σ(*I*)
*R*_int_ = 0.059

#### Refinement

*R*[*F* ^2^ > 2σ(*F* ^2^)] = 0.055
*wR*(*F* ^2^) = 0.107
*S* = 0.94
2475 reflections
154 parameters
3 restraints
H atoms treated by a mixture of independent and constrained refinement
Δρ_max_ = 0.20 e Å^−3^
Δρ_min_ = −0.33 e Å^−3^

### 

Data collection: *CrystalClear* (Rigaku, 2005 ▶); cell refinement: *CrystalClear*; data reduction: *CrystalClear*; program(s) used to solve structure: *SHELXTL* (Sheldrick, 2008 ▶); program(s) used to refine structure: *SHELXTL*; molecular graphics: *SHELXTL*; software used to prepare material for publication: *SHELXTL*.

## Supplementary Material

Crystal structure: contains datablock(s) I, global. DOI: 10.1107/S1600536812019149/yk2053sup1.cif

Structure factors: contains datablock(s) I. DOI: 10.1107/S1600536812019149/yk2053Isup2.hkl

Supplementary material file. DOI: 10.1107/S1600536812019149/yk2053Isup3.cml

Additional supplementary materials:  crystallographic information; 3D view; checkCIF report

## Figures and Tables

**Table 1:** Hydrogen-bond geometry (Å, °)

*D*—H⋯*A*	*D*—H	H⋯*A*	*D*⋯*A*	*D*—H⋯*A*
O4—H4*C*⋯O2^i^	0.82 (2)	1.95 (2)	2.754 (3)	167 (3)
O4—H4*B*⋯O2^ii^	0.82 (2)	1.92 (2)	2.730 (3)	166 (3)
N1—H1*D*⋯O1^iii^	0.86	2.00	2.768 (3)	149
N2—H2*B*⋯O4	0.86	1.78	2.628 (3)	169

Symmetry codes: (i) 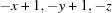; (ii) 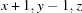; (iii) 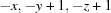.

**Fig. 1.**
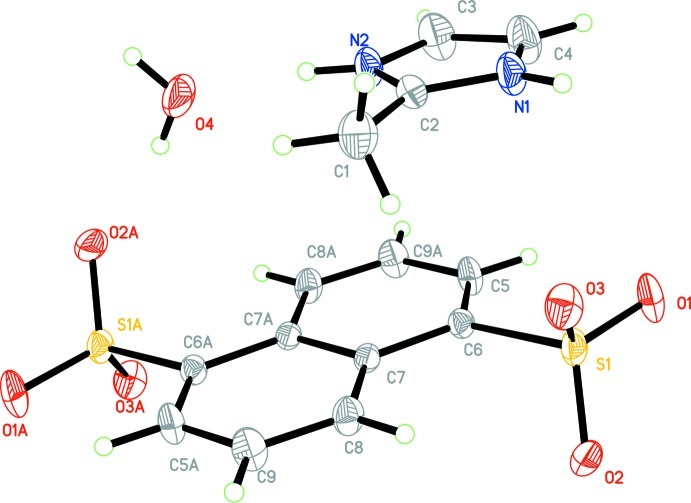
The structure of the title compound, with the atomic numbering scheme. Displacement ellipsoids are drawn at the 30% probability level. Atoms labelled with suffix A are generated by the symmetry operator (1 - x, 1 - y, -z).

**Fig. 2.**
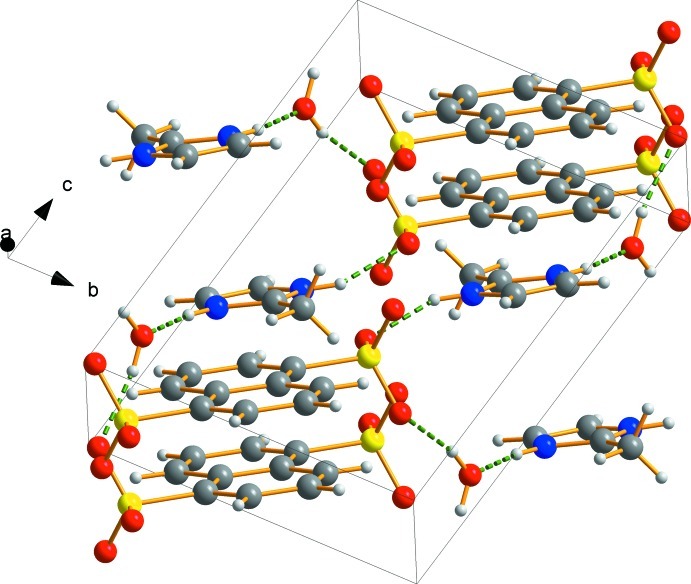
A view of the packing of the title compound, stacking along the a axis. Dashed lines indicate hydrogen bonds.

## supplementary crystallographic information

### Comment

The compounds exhibiting the dielectric-ferroelectric phase transition constitute an interesting class of materials, comprising organic and metal-organic coordination compounds, organic-inorganic hybrids and organic salts (Ye *et al.*, 2009; Zhang *et al.*, 2009). Unfortunately, the temperature dependence of dielectric constant of the title compound indicates that the permittivity is temperature-independent below the melting point (388 - 389 K) of the compound. Herein we descibe the crystal structure of this compound.

The asymmetric unit of the title compound consists of a 2-methylimidazolium cation, a half of naphthalene-1,5-disulfonate anion and a water molecule (Fig. 1). The cations, anions and water molecules are connected by N—H···O and O—H···O hydrogen bonds, which form the layers parallel to the (1 1 1) plane (Fig. 2 and Table 1).

### Experimental

The title compound was obtained by the addition of naphthalene-1,5-disulfonic acid (2.88 g, 0.01 mol) to a solution of 2-methylimidazole (1.6 g, 0.02 mol) in water. Good quality single crystals were obtained by slow evaporation after two days (the chemical yield is 45%).

### Refinement

All H atoms attached to C and N atoms were placed in geometrically idealized positions and constrained to ride on their parent atoms with C—H = 0.93 Å–0.96 Å and N—H = 0.86 Å and with *U*_iso_(H) = 1.5*U*_eq_(C,N) Bond lengths O—H were restrained to 0.82 (2) Å.

### Crystal data

**Table:**

2C_4_H_7_N_2_^+^·C_10_H_6_O_6_S_2_^2^^−^·2H_2_O	*Z* = 1
*M**_r_* = 488.53	*F*(000) = 256
Triclinic, *P*1	*D*_x_ = 1.500 Mg m^−^^3^
Hall symbol: -P 1	Mo *K*α radiation, λ = 0.71073 Å
*a* = 7.1301 (14) Å	Cell parameters from 3638 reflections
*b* = 8.1773 (16) Å	θ = 3.0–27.5°
*c* = 9.970 (2) Å	µ = 0.30 mm^−^^1^
α = 75.58 (3)°	*T* = 293 K
β = 75.10 (3)°	Block, colourless
γ = 80.34 (3)°	0.23 × 0.22 × 0.18 mm
*V* = 540.7 (2) Å^3^	

### Data collection

**Table:**

Rigaku SCXmini diffractometer	2475 independent reflections
Radiation source: fine-focus sealed tube	1490 reflections with *I* > 2σ(*I*)
Graphite monochromator	*R*_int_ = 0.059
Detector resolution: 13.6612 pixels mm^-1^	θ_max_ = 27.5°, θ_min_ = 3.3°
ω scans	*h* = −9→9
Absorption correction: multi-scan (*CrystalClear*; Rigaku, 2005)	*k* = −10→10
*T*_min_ = 0.933, *T*_max_ = 0.947	*l* = −12→12
5695 measured reflections	

### Refinement

**Table:**

Refinement on *F*^2^	Primary atom site location: structure-invariant direct methods
Least-squares matrix: full	Secondary atom site location: difference Fourier map
*R*[*F*^2^ > 2σ(*F*^2^)] = 0.055	Hydrogen site location: inferred from neighbouring sites
*wR*(*F*^2^) = 0.107	H atoms treated by a mixture of independent and constrained refinement
*S* = 0.94	*w* = 1/[σ^2^(*F*_o_^2^) + (0.0396*P*)^2^] where *P* = (*F*_o_^2^ + 2*F*_c_^2^)/3
2475 reflections	(Δ/σ)_max_ = 0.005
154 parameters	Δρ_max_ = 0.20 e Å^−^^3^
3 restraints	Δρ_min_ = −0.33 e Å^−^^3^

### Special details

**Table:**

Geometry. All s.u.'s (except the s.u. in the dihedral angle between two l.s. planes) are estimated using the full covariance matrix. The cell s.u.'s are taken into account individually in the estimation of s.u.'s in distances, angles and torsion angles; correlations between s.u.'s in cell parameters are only used when they are defined by crystal symmetry. An approximate (isotropic) treatment of cell s.u.'s is used for estimating s.u.'s involving l.s. planes.
Refinement. Refinement of *F*^2^ against ALL reflections. The weighted *R*-factor *wR* and goodness of fit *S* are based on *F*^2^, conventional *R*-factors *R* are based on *F*, with *F* set to zero for negative *F*^2^. The threshold expression of *F*^2^ > σ(*F*^2^) is used only for calculating *R*-factors(gt) *etc*. and is not relevant to the choice of reflections for refinement. *R*-factors based on *F*^2^ are statistically about twice as large as those based on *F*, and *R*- factors based on ALL data will be even larger.

### Fractional atomic coordinates and isotropic or equivalent isotropic displacement parameters (Å^2^)

**Table:**

	*x*	*y*	*z*	*U*_iso_*/*U*_eq_	
O4	0.7815 (4)	0.0540 (4)	0.1408 (3)	0.0873 (9)	
C9	0.7423 (4)	0.6967 (3)	−0.0199 (3)	0.0419 (7)	
H9A	0.8089	0.7809	−0.0115	0.050*	
S1	0.13194 (10)	0.66733 (9)	0.23893 (7)	0.0342 (2)	
O3	0.2455 (3)	0.6595 (2)	0.33926 (19)	0.0438 (5)	
O2	0.1153 (3)	0.8363 (2)	0.1501 (2)	0.0481 (5)	
C7	0.4534 (3)	0.5560 (3)	0.0451 (2)	0.0255 (5)	
C6	0.2599 (3)	0.5340 (3)	0.1233 (2)	0.0271 (6)	
O1	−0.0547 (2)	0.6045 (2)	0.3027 (2)	0.0501 (5)	
N2	0.4574 (3)	0.1263 (3)	0.3230 (2)	0.0410 (6)	
H2B	0.5580	0.0903	0.2647	0.049*	
N1	0.2739 (3)	0.2647 (3)	0.4672 (2)	0.0438 (6)	
H1D	0.2318	0.3366	0.5212	0.053*	
C2	0.4508 (4)	0.2493 (3)	0.3875 (3)	0.0342 (6)	
C8	0.5575 (4)	0.6821 (3)	0.0549 (3)	0.0349 (6)	
H8A	0.4977	0.7567	0.1140	0.042*	
C5	0.1658 (4)	0.4121 (3)	0.1091 (3)	0.0375 (7)	
H5C	0.0378	0.4008	0.1597	0.045*	
C3	0.2826 (4)	0.0645 (4)	0.3619 (3)	0.0571 (9)	
H3A	0.2496	−0.0228	0.3308	0.069*	
C1	0.6089 (4)	0.3484 (4)	0.3718 (3)	0.0500 (8)	
H1A	0.6461	0.3283	0.4610	0.075*	
H1B	0.5664	0.4669	0.3429	0.075*	
H1C	0.7188	0.3156	0.3012	0.075*	
C4	0.1682 (5)	0.1513 (4)	0.4521 (3)	0.0565 (9)	
H4A	0.0386	0.1372	0.4970	0.068*	
H4B	0.876 (4)	−0.014 (4)	0.158 (3)	0.083 (13)*	
H4C	0.796 (4)	0.096 (4)	0.056 (2)	0.069 (11)*	

### Atomic displacement parameters (Å^2^)

**Table:**

	*U*^11^	*U*^22^	*U*^33^	*U*^12^	*U*^13^	*U*^23^
O4	0.0703 (17)	0.107 (2)	0.0409 (17)	0.0527 (15)	0.0101 (13)	−0.0005 (15)
C9	0.0391 (16)	0.0428 (18)	0.0478 (19)	−0.0129 (13)	0.0012 (14)	−0.0228 (14)
S1	0.0311 (4)	0.0392 (4)	0.0294 (4)	0.0027 (3)	0.0011 (3)	−0.0144 (3)
O3	0.0459 (11)	0.0558 (13)	0.0349 (11)	0.0010 (9)	−0.0114 (10)	−0.0213 (9)
O2	0.0517 (12)	0.0362 (12)	0.0419 (12)	0.0143 (9)	0.0011 (10)	−0.0067 (9)
C7	0.0236 (13)	0.0277 (14)	0.0237 (14)	0.0011 (10)	−0.0050 (11)	−0.0058 (10)
C6	0.0269 (13)	0.0310 (14)	0.0219 (14)	−0.0001 (11)	−0.0032 (11)	−0.0075 (11)
O1	0.0318 (11)	0.0682 (14)	0.0485 (13)	−0.0118 (9)	0.0141 (9)	−0.0293 (10)
N2	0.0377 (13)	0.0429 (15)	0.0381 (14)	−0.0014 (11)	0.0069 (11)	−0.0192 (11)
N1	0.0426 (14)	0.0420 (15)	0.0422 (15)	−0.0042 (11)	0.0096 (12)	−0.0209 (11)
C2	0.0340 (15)	0.0357 (16)	0.0281 (16)	−0.0021 (12)	−0.0008 (13)	−0.0059 (12)
C8	0.0340 (15)	0.0366 (16)	0.0367 (16)	−0.0023 (12)	−0.0023 (13)	−0.0194 (12)
C5	0.0231 (14)	0.0485 (18)	0.0402 (17)	−0.0082 (12)	0.0031 (13)	−0.0162 (13)
C3	0.057 (2)	0.048 (2)	0.066 (2)	−0.0220 (16)	0.0103 (18)	−0.0273 (17)
C1	0.0377 (17)	0.060 (2)	0.055 (2)	−0.0064 (15)	−0.0092 (15)	−0.0183 (16)
C4	0.0457 (18)	0.052 (2)	0.068 (2)	−0.0221 (15)	0.0142 (17)	−0.0241 (17)

### Geometric parameters (Å, º)

**Table:**

O4—H4B	0.824 (17)	N2—H2B	0.8600
O4—H4C	0.816 (16)	N1—C2	1.311 (3)
C9—C8	1.346 (3)	N1—C4	1.347 (3)
C9—C5^i^	1.382 (3)	N1—H1D	0.8600
C9—H9A	0.9300	C2—C1	1.451 (3)
S1—O3	1.4237 (18)	C8—H8A	0.9300
S1—O1	1.4392 (19)	C5—C9^i^	1.382 (3)
S1—O2	1.4483 (19)	C5—H5C	0.9300
S1—C6	1.761 (2)	C3—C4	1.313 (4)
C7—C8	1.402 (3)	C3—H3A	0.9300
C7—C7^i^	1.410 (4)	C1—H1A	0.9600
C7—C6	1.415 (3)	C1—H1B	0.9600
C6—C5	1.345 (3)	C1—H1C	0.9600
N2—C2	1.310 (3)	C4—H4A	0.9300
N2—C3	1.351 (3)		
			
H4B—O4—H4C	112 (2)	N2—C2—N1	106.4 (2)
C8—C9—C5^i^	120.6 (2)	N2—C2—C1	126.3 (2)
C8—C9—H9A	119.7	N1—C2—C1	127.3 (2)
C5^i^—C9—H9A	119.7	C9—C8—C7	121.1 (2)
O3—S1—O1	113.33 (12)	C9—C8—H8A	119.5
O3—S1—O2	111.00 (12)	C7—C8—H8A	119.5
O1—S1—O2	112.51 (12)	C6—C5—C9^i^	120.6 (2)
O3—S1—C6	107.66 (11)	C6—C5—H5C	119.7
O1—S1—C6	106.15 (12)	C9^i^—C5—H5C	119.7
O2—S1—C6	105.65 (11)	C4—C3—N2	106.8 (3)
C8—C7—C7^i^	118.6 (3)	C4—C3—H3A	126.6
C8—C7—C6	123.1 (2)	N2—C3—H3A	126.6
C7^i^—C7—C6	118.3 (3)	C2—C1—H1A	109.5
C5—C6—C7	120.8 (2)	C2—C1—H1B	109.5
C5—C6—S1	117.62 (19)	H1A—C1—H1B	109.5
C7—C6—S1	121.57 (18)	C2—C1—H1C	109.5
C2—N2—C3	109.9 (2)	H1A—C1—H1C	109.5
C2—N2—H2B	125.1	H1B—C1—H1C	109.5
C3—N2—H2B	125.1	C3—C4—N1	107.1 (3)
C2—N1—C4	109.9 (2)	C3—C4—H4A	126.5
C2—N1—H1D	125.1	N1—C4—H4A	126.5
C4—N1—H1D	125.1		

Symmetry code: (i) −*x*+1, −*y*+1, −*z*.

### Hydrogen-bond geometry (Å, º)

**Table:**

*D*—H···*A*	*D*—H	H···*A*	*D*···*A*	*D*—H···*A*
O4—H4*C*···O2^i^	0.82 (2)	1.95 (2)	2.754 (3)	167 (3)
O4—H4*B*···O2^ii^	0.82 (2)	1.92 (2)	2.730 (3)	166 (3)
N1—H1*D*···O1^iii^	0.86	2.00	2.768 (3)	149
N2—H2*B*···O4	0.86	1.78	2.628 (3)	169

Symmetry codes: (i) −*x*+1, −*y*+1, −*z*; (ii) *x*+1, *y*−1, *z*; (iii) −*x*, −*y*+1, −*z*+1.
